# Development of the Preferred Components for Co-Design in Research Guideline and Checklist: Protocol for a Scoping Review and a Modified Delphi Process

**DOI:** 10.2196/50463

**Published:** 2023-10-30

**Authors:** Sarah EP Munce, Carolyn Steele Gray, Beverley Claire Pomeroy, Mark Bayley, Kristina Marie Kokorelias, Dorothy Luong, Elaine Biddiss, Trish Cave, Peter Bragge, Carolyn A Chew-Graham, Heather Colquhoun, Ann Dadich, Katie N Dainty, Mark Elliott, Patrick Feng, Jodeme Goldhar, Clayon B Hamilton, Gillian Harvey, Monika Kastner, Anita Kothari, Joe Langley, Lianne Jeffs, Daniel Masterson, Michelle LA Nelson, Laure Perrier, John Riley, Kate Sellen, Emily Seto, Robert Simpson, Sophie Staniszewska, Vasanthi Srinivasan, Sharon E Straus, Andrea C Tricco, Kerry Kuluski

**Affiliations:** 1 KITE Toronto Rehabilitation Institute University Health Network Toronto, ON Canada; 2 Department of Occupational Science and Occupational Therapy Temerty Faculty of Medicine University of Toronto Toronto, ON Canada; 3 Institute of Health Policy, Management and Evaluation University of Toronto Toronto, ON Canada; 4 Rehabilitation Sciences Institute Temerty Faculty of Medicine University of Toronto Toronto, ON Canada; 5 Lunenfeld-Tanenbaum Research Institute Sinai Health Toronto, ON Canada; 6 Division of Physical Medicine and Rehabilitation Temerty Faculty of Medicine University of Toronto Toronto, ON Canada; 7 Sinai Health and University Health Network Geriatrics Toronto, ON Canada; 8 Institute of Biomedical Engineering University of Toronto Toronto, ON Canada; 9 Holland Bloorview Kids Rehabilitation Hospital Toronto, ON Canada; 10 Collabforge Victoria Australia; 11 Monash Sustainable Development Institute Evidence Review Service BehaviourWorks Australia Monash University, Clayton Campus Melbourne, Victoria Australia; 12 School of Medicine Faculty of Medicine and Health Sciences Keele University Newcastle United Kingdom; 13 School of Business Western Sydney University Sydney Australia; 14 North York General Hospital Toronto, ON Canada; 15 Ontario College of Art & Design University Toronto, ON Canada; 16 International Foundation for Integrated Care Toronto, ON Canada; 17 Faculty of Health Sciences Simon Fraser University Vancouver, BC Canada; 18 Design Studies Department of Art & Design University of Alberta Edmonton, AB Canada; 19 School of Health Studies Western University London, ON Canada; 20 Lab4Living Sheffield Hallam University Sheffield United Kingdom; 21 Jönköping Academy for Improvement of Health and Welfare Sweden School of Health and Welfare Jönköping University Jönköping Sweden; 22 Dalla Lana School of Public Health University of Toronto Toronto, ON Canada; 23 Ontario Strategy for Patient-Oriented Research SUPPORT Unit Toronto, ON Canada; 24 Centre for Digital Therapeutics University Health Network Toronto, ON Canada; 25 Institute of Health and Wellbeing University of Glasgow Scotland United Kingdom; 26 Health Sciences Warwick Medical School University of Warwick Coventry United Kingdom; 27 Li Ka Shing Institute of St. Michael’s Hospital Unity Health Toronto, ON Canada; 28 Queen’s Collaboration for Health Care Quality: A JBI Centre of Excellence Queen’s University School of Nursing Queen's University Kingston, ON Canada; 29 Institute for Better Health Trillium Health Partners Mississauga, ON Canada

**Keywords:** co-design, reporting, guidelines, complex health intervention, scoping review, consensus

## Abstract

**Background:**

There is increasing evidence that co-design can lead to more engaging, acceptable, relevant, feasible, and even effective interventions. However, no guidance is provided on the specific designs and associated methods or methodologies involved in the process. We propose the development of the Preferred Components for Co-design in Research (PRECISE) guideline to enhance the consistency, transparency, and quality of reporting co-design studies used to develop complex health interventions.

**Objective:**

The aim is to develop the first iteration of the PRECISE guideline. The purpose of the PRECISE guideline is to improve the consistency, transparency, and quality of reporting on studies that use co-design to develop complex health interventions.

**Methods:**

The aim will be achieved by addressing the following objectives: to review and synthesize the literature on the models, theories, and frameworks used in the co-design of complex health interventions to identify their common elements (components, values or principles, associated methods and methodologies, and outcomes); and by using the results of the scoping review, prioritize the co-design components, values or principles, associated methods and methodologies, and outcomes to be included in the PRECISE guideline.

**Results:**

The project has been funded by the Canadian Institutes of Health Research.

**Conclusions:**

The collective results of this project will lead to a ready-to-implement PRECISE guideline that outlines a minimum set of items to include when reporting the co-design of complex health interventions. The PRECISE guideline will improve the consistency, transparency, and quality of reports of studies. Additionally, it will include guidance on how to enact or enable the values or principles of co-design for meaningful and collaborative solutions (interventions). PRECISE might also be used by peer reviewers and editors to improve the review of manuscripts involving co-design. Ultimately, the PRECISE guideline will facilitate more efficient use of new results about complex health intervention development and bring better returns on research investments.

**International Registered Report Identifier (IRRID):**

PRR1-10.2196/50463

## Introduction

Health research can often be immaterial when it focuses on questions and outcomes that have limited relevance to the individuals who will ultimately use the research findings, such as patients and clinicians [1]. A scoping study conducted by Oliver and Gray [2] in 2006 found that out of 334 studies, only 9 compared the priorities of researchers with those of patients or clinicians in terms of research and outcomes for assessing treatment effects. If end users, including patients and clinicians, are excluded from the process of establishing research priorities, such as in the selection of research areas or intervention development, irrelevant health research results and interventions might be generated [3].

To address this issue, there has been a notable increase in patient and public involvement (PPI) in health research [4-11]. Acknowledging the significance of incorporating the viewpoints and firsthand knowledge of patients, families, clinicians, and policymakers, many funding agencies have made patient-oriented activities, also referred to as PPI, obligatory [12-14]. According to the Canadian Institutes of Health Research (CIHR), PPI refers to “a continuum of research, from initial studies in humans to comparative effectiveness and outcomes research, and the integration of this research into the health system and clinical practice” [15]. Thus, PPI involves actively involving patients, carers, and members of the public in various stages of research, such as setting priorities, designing studies, collecting and interpreting data, designing interventions, and disseminating findings [14,16,17]. This shift towards PPI signifies a paradigm shift in the research landscape, emphasizing the significance of collaborative and inclusive approaches to better address the needs and preferences of the communities being served [13,18,19]. Thus, researchers are challenged to “do... [PPI] right” [20].

To enact PPI, researchers have used various collaborative and participatory methods and designs [21]. One approach that has garnered significant attention and momentum is co-design, as a specific application and democratization of design that originated from the field of participatory design [22,23]. Co-design entails a process of collective creativity that spans the entire lifecycle of a design project [24]. This approach embraces creativity and flexibility to foster reflective feedback throughout the project [22]. Unlike the traditional expert-led approach, where interventions are designed “for” users, co-design emphasizes designing interventions “with” or “by” users. Thus, co-design approaches aim to engage patients and other knowledge users in meaningful and reciprocal collaborations, fostering ownership, empowerment, and shared responsibility [25,26]. During co-design, active collaboration occurs among researchers, designers, developers, and users as experts in their experiences [27,28]. Co-design involves cooperatively exploring and communicating needs and devising solutions as a team [29,30]. Co-design aims for better design based on a richer, deeper understanding of what users know and experience [29]. Co-design in the domain of health services research, specifically in this case, involves multiple end-users, including researchers, designers, developers, and users, including health care workers and patients as experts who contribute their collective input for the purpose of developing a complex health intervention [24,27,29].

There is increasing evidence that co-design can lead to more engaging, acceptable, relevant, feasible, and even effective interventions [25,31]. A systematic review [8] reported examples of practice change from introducing co-designed outputs [31]. Changes included improved consistency in clinician assessment and identification of patient problems that were previously missed [32], revised clinical pathways [33], fewer hospital visits and admissions [32], and a reduction in the number of patients who failed to attend appointments [34]. Additional beneficial outcomes, such as patient satisfaction, were either demonstrated or perceived to be possible [35].

When practicing co-design, most researchers agree that co-design processes begin with understanding users’ needs and behaviors and developing concepts that are tested and adapted in simple, fast, and low-cost ways [29,30]. For example, Boyd and colleagues [36] describe a 6-step process that includes engaging, planning, exploring, developing, deciding, and changing. However, there is considerable variation in how co-design is defined and practiced, ranging from feedback and consultation to user testing to web-based collaboration or user research and workshops [25]. Moreover, in practice, the terms co-design, cocreation, and coproduction are often used interchangeably and adopted and described inadequately, collectively termed “coapproaches” [23,31,37-39]. A recent overview of 23 reviews [25] concluded that, while co-design in health care appears to be widely used to develop complex health interventions, it is seldom described or evaluated in detail. The authors further concluded that realizing the potential of research-based co-design might require clearer and more consistent terminology and better reporting of the activities involved [25]. While some of the fundamentals of coapproaches have been described, for example, sharing power, including all perspectives and skills, respecting and valuing all knowledge, reciprocity, and building and maintaining relationships [40], there is limited consistency in understanding how to co-design complex health interventions and report them. Complex health interventions are defined as any effort, activity, or combination of program components that independently and interdependently improve health outcomes. Complex health interventions can be delivered in many settings, including health services, schools, local communities, or national populations. They can be delivered by a variety of individuals, including health care, social care, and public health practitioners, as well as professionals working outside of the health care sector, such as peers [41-43]. An example of a complex health intervention is a self-management program involving educational materials or resources and motivational enhancement delivered via weekly interactions with a peer. Complex health interventions do not include the development of medicines and any invasive interventions (eg, medical or surgical procedures).

In response to the reporting challenges specific to PPI in research, the Guidance for Reporting Involvement of Patients and the Public (GRIPP) [44] and GRIPP2 [45] were developed**.** The use of reporting guidelines can enhance transparency in the methodology presented in research reports and improve the adoption of research findings [45,46]. Reporting guidelines refer to a prescribed set of essential elements that should be included in co-design research reports [47]. These guidelines can be in the form of a checklist, flow diagram, or explicit text to assist authors in accurately reporting specific types of research [47]. The development of guidelines like these follows a well-defined methodology [47]. Hence, reporting checklists have dual applications: they can be used proactively to guide the research design, or retrospectively to evaluate the quality of reporting. Indeed, systematic reviewers have found the endorsement and implementation of guidelines by journals can improve reporting [46,48]. Ultimately, reporting guidelines contribute to the more effective use of new research findings and yield enhanced returns on research investments [46,48].

Despite the contribution of GRIPP2 [45], its guidance is generic and not focused on a particular approach or methodology—this leaves room to develop reporting guidance for particular approaches, including co-design approaches involving patients and other end-users to develop complex health interventions. Additionally, no guidance is provided on the specific designs and associated methods or methodologies involved in the process (eg, needs assessment via qualitative methods to clarify and address gaps in intervention preferences). Furthermore, GRIPP2 does not include guidance on the reporting of values or principles associated with meaningful co-design and how they are enacted or enabled. Thus, new guidelines are needed to promote consistent, transparent, and quality reporting of co-design studies to develop complex health intervention development.

We propose the development of the Preferred Components for Co-design in Research (PRECISE) guideline to enhance the consistency, transparency, and quality of reporting co-design studies used to develop complex health interventions. This aim will be realized by addressing the following objectives: to conduct a scoping review on the models, theories, and frameworks used to co-design complex health interventions to identify their common elements (components, values or principles, associated methods and methodologies, and outcomes); and to use these results to prioritize the co-design components, values or principles, associated methods and methodologies, and outcomes to be included in the PRECISE guideline using a modified Delphi process.

## Methods

### Study Design

The development of the PRECISE guideline will follow the methodological framework for developing reporting guidelines, as outlined by the Enhancing the Quality and Transparency of Health Research (EQUATOR) Network [49], and the Guidance for Developers of Health Research Reporting Guidelines [20].

### Integrated Knowledge Translation Panel

An integrated knowledge translation (iKT) panel has been formed to codevelop the research protocol, including informing the search strategy, reviewing preliminary results, and planning knowledge dissemination strategies. The iKT panel includes patient partners, caregivers, health care administrators, and journal editors. The formation of the iKT panel aims to codevelop the research protocol, which includes ensuring patient-based data representation in journals, informing the search strategy, reviewing preliminary results, and planning knowledge dissemination strategies, all while fostering a brave space that promotes trust, inclusion, and respect among patient partners, caregivers, health care administrators, and journal editors involved in the process. We will use the reflective exercise titled “SPOREA Reflective EDI Exercise” to promote dialogue and understanding around equity, diversity, and inclusion (EDI) topics amongst all iKT panel members [50]. We will ensure that discussions are conducted in a manner that respects diverse perspectives and experiences. We will compensate patients for their time and expertise according to mutually agreed-upon guidelines, which will be informed by the SPOR Evidence Alliance’s policies and procedures [51].

### Phase 1: Scoping Review on Models, Theories, and Frameworks in the Co-Design of Complex Health Interventions

#### Study Design

The methodology for this scoping review was developed following the methodological frameworks of JBI [52] and Khalil and colleagues’ [53] suggestions. The protocol for this scoping review was registered on the Open Science Framework Register on June 12, 2023 (osf.io/prd3t). The protocol was guided by and reported according to the Preferred Reporting Items for Systematic Review and Meta-Analysis Protocols (PRISMA-P) [54]. The results of the review will be reported using the Preferred Reporting Items for Systematic Reviews and Meta-Analysis Extension for Scoping Reviews (PRISMA-ScR) checklist [55].

#### Stage 1: Developing a Search Strategy

An information specialist and health science librarian will create and draft the search strategy using OVID Medline in consultation with the research team. The Population, Concept, and Context framework will be used to guide the search strategy (population: model, theory, or frameworks; concept: co-design studies; context: complex health interventions). Literature search strategies using medical subject headings and text words related to co-design, cocreation, and coproduction and models, theories, and frameworks will be developed. This preliminary search strategy is based on the published search strategies of previous reviews [25,31]. The final search strategy will combine structure database-specific subject headings (as available) and keywords or synonyms. The final search strategy will also undergo peer review using the Peer Review of Electronic Search Strategies (PRESS) statement checklist [56].

The following databases will be searched: Medline, CINAHL, Embase, PsycINFO, ACM Digital Library, and Cochrane Central Register of Controlled Trials. Hand-searching of design-specific publications will also be performed (eg, *Design for Health*, *HERD: Health Environments Research & Design Journal*, and *Health Design*). To help capture any relevant literature, we will also search the reference lists of included studies and those of relevant systematic reviews. The searches will not be limited by study design or languages. The search will capture literature from 1972 to present, consistent with when the term co-design first originated [23,57]. We will also search the gray literature in specialized databases like OpenGrey, Grey Literature Report, and GreyNet International; platforms like arXiv, bioRxiv, and SSRN; and databases like ProQuest Dissertations and Theses. We will search for conference abstracts (full papers) in specialized co-design in health conference proceedings (ie, D4H Proceedings).

#### Stage 2: Evidence Screening and Selection

All primary studies that have applied a model, theory, or framework for the purpose of co-designing a complex health intervention will be eligible for inclusion. We define a model as the essential elements or variables of a phenomenon or a specific aspect of a phenomenon; a theory as “a set of analytical principles or statements designed to structure our observation, understanding, and explanation of the world;” and a framework as an explanation of a phenomenon by organizing it into a collection of descriptive categories and the relationships between them [58]. We focus on models, theories, and frameworks to identify key components, actions, and mechanisms.

All articles with primary study designs will be included (eg, experimental, quasi-experimental, observational, qualitative, mixed, and multiple methods). Systematic reviews, meta-analyses, editorials, commentaries, and nonspecific conference proceedings will be excluded to focus on including primary results and not preliminary findings or ongoing research; however, the reference lists of relevant reviews will be hand searched for relevant articles.

References for all included studies will be uploaded and managed through EndNote (Niles & Associates) [59] and duplicates will be removed before importing sources into Covidence (developed by an Australian not-for-profit company) [60]. To increase the reliability, a pilot test of the level 1 (title and abstract) screening form based on the criteria outlined above will be conducted on a random sample of approximately 50 papers by the entire team. The descriptions of the eligibility criteria will be revised if deemed necessary by the team or if a low agreement (ie, <70%) [61] is observed to improve the consistent application of the selection criteria**.** Our agreement will be defined by Cohen κ [62]. All screening (ie, levels 1 and 2, full-text) will occur in duplicate and independently. For level 1 screening, reviewers will screen the titles and abstracts for inclusion using Covidence. For level 2, the full text of potentially relevant articles will then be collected and screened to determine final inclusion. A pilot test of the level 2 screening will also be performed on approximately 25% of the articles, similar to the process for level 1 screening. When necessary, another reviewer knowledgeable in the research area will be available to resolve conflicts. For studies that are excluded at level 2, the reason for exclusion will be recorded. Evidence screening will be managed using Covidence software [60]. Members of the research team, including members of the iKT panel, will conduct the screening.

#### Stage 3: Data Extraction

A standardized data extraction form will be developed by the research team, in consultation with the JBI manual data extraction suggestions [52] and the recommendations for the extraction, analysis, and presentation of results in scoping reviews [63]. Extracted data will include study characteristics (eg, year of publication and study design), participant population characteristics (eg, medical condition, age, race, sex, gender, and gender-related variables such as education and health literacy), types of health interventions (eg, in-person support program and apps), and details of the models, theories, frameworks (eg, name, reference, date of initial development, components, values or principles (and how they were enabled or enacted), associated methods and methodologies) that have been used in the co-design of complex health interventions. We will also collect data on how end users were engaged throughout the various stages of the co-design process and outcomes achieved as well as the results of the study. Additional categories will likely be identified through the completion of the search and through discussions with the research team.

To ensure all relevant results are extracted, 2 research assistants will conduct a pilot extraction by trialing the extraction template for 2 to 3 articles. All data will then be extracted in duplicate by 2 independent reviewers. Discrepancies in the extracted data will be discussed and resolved by the 2 reviewers. Quality or risk of bias will not be assessed, as this is not required in scoping reviews [55].

#### Stage 4: Data Analysis

The bibliographic data from this scoping review will be quantitatively summarized using numerical counts (eg, number of studies from Canada, the United States) and qualitatively using content analysis [64]. These data will be analyzed or coded manually. The data will be grouped by the main components of the model, theories, frameworks, values or principles (and how they were enacted), associated methods (eg, one-on-one interviews), and methodologies. We will also synthesize data on how end users were engaged throughout the various stages of the co-design process, the types of outcomes collected, as well as results. Depending on the included articles, subgroup analyses might be conducted by, for instance, complex health intervention type, sex, gender-related variables, as well as the other PROGRESS-Plus characteristics (eg, race, ethnicity, culture, language, education) [65]. If feasible, we will contact the study authors of the included studies to confirm that all the data collected were included in the article (eg, not excluded due to word count limitations of a journal).

#### Dissemination

The results of this scoping review will be used to inform the surveys for the Delphi discussions that will be implemented in phase 2, as well as the draft PRECISE guideline. Findings from the scoping review will help to identify which components, values or principles, associated methods and methodologies, and outcomes will be included in the guideline. Other outputs from the scoping review will include a peer-reviewed publication of the results, as well as a newsletter and webinar to promote engagement for phase 2.

### Phase 2: Modified Delphi Process to Prioritize Items for the PRECISE Guideline

#### Study Design

As consensus-building processes are crucial in developing a reporting guideline as per the EQUATOR process [49], the prioritization of the PRECISE guideline items will occur using a modified Delphi process [47]. A modified Delphi process refers to a variation or adaptation of the traditional Delphi method. The Delphi method is a structured and iterative process used to gather insights and perspectives from a panel of experts to reach a consensus or make informed decisions [47,66]. The Delphi process involves repeated surveys to collect previously unknown information from a group of individuals with expertise in a particular area. Participants in a Delphi process are usually given a large number of items that need to be rated or ranked. Consistent with the methods used to develop other reporting guidelines [55], we will conduct this process over 3 rounds. The succeeding iterations of each round of the Delphi process are intended to decrease the number of items based on analyses of participant responses and denote the consensus of these experts.

#### Identification and Recruitment of Participants for the Modified Delphi Process and Web-Based Consensus Meeting

A multinational, multidisciplinary, and panel of experts specializing in co-design for the development of complex health interventions will be invited to engage in the modified Delphi process. This panel will consist of individuals from various geographical locations, diverse knowledge user types (including patients, caregivers, and members of the public), and a range of research backgrounds and personal characteristics such as sex, gender, and ethnicity. The experts within this panel will actively contribute their insights and recommendations, particularly emphasizing the involvement of specific patient and caregiver knowledge users.

Given the breadth and diversity of expertise and lived experience required for this study, we will use a purposive and snowball sampling strategy to achieve the recommended minimum sample size of 100-150, consistent with other reporting guideline initiatives [67].

Invitations will be extended to potential expert participants via email, accompanied by a comprehensive explanation of the initiative. To ensure inclusivity, individuals facing technological barriers will be offered assistance to facilitate their participation. This may involve conducting surveys over the phone or providing participants with necessary devices such as tablets, along with any required training in using the technology.

#### Data Collection

The round 1 survey will be developed based on the findings of the scoping review. Findings from the scoping review will generate an initial list of PRECISE guideline items that will be reviewed by the researchers and iKT panel. The survey tool will ask participants to rank key concepts in terms of relevance, importance, and comment on the definition of the concept. Pilot testing will be conducted to ensure the content is clear and comprehensible, and necessary revisions will be made before distribution. Participants who complete the round 1 survey will be eligible to participate in the round 2 survey. Likewise, completion of the round 2 survey will determine eligibility for the round 3 survey. Participants in round 2 will be presented with the condensed list of concepts and asked to rank order from most to least important. As with round 1, experts will be asked to comment on the particular concepts listed and add any other categories not identified. This will be repeated for round 3.

During all rounds, participants will be asked to provide demographic and descriptive information, such as sex, gender identity, race, expert participant type, and career stage (if applicable). The web-based survey will be administered using Qualtrics software (Qualtrics XM) for all 3 rounds. This will last 4 weeks, and reminder emails will be sent every 7 days after the initial invitation to encourage participation. Each survey is estimated to take approximately 20 minutes to complete.

Participants will be requested to rate their agreement with the inclusion of each proposed guideline item using a 7-point Likert scale, ranging from “entirely disagree” to “entirely agree.” Surveys will be conducted exclusively in English. Furthermore, each survey item will include an optional text box for participants to provide comments, including suggestions for additions, deletions, aggregations, or refinements of items. This allows for the opportunity to modify and retain items based on participant feedback.

Upon joining the study, participants will be given an exclusive research code for identification purposes. A secure record containing the association between participants’ names and their respective research codes will be maintained on a protected network. Access to this record will be restricted solely to the research administrator responsible for supporting the study.

#### Analysis

Demographic and descriptive information provided by participants will be analyzed by calculating proportions expressed as percentages. This information will include factors such as sex, gender identity, race, knowledge user type, and career stage if applicable.

Throughout all 3 rounds of the survey, a consensus threshold of 80% agreement will be applied to each guideline item. This means that for an item to be considered as achieving consensus and being kept in the PRECISE guideline, at least 80% of participants must select values of 6 or 7 (mostly or entirely agree with its inclusion) on the Likert scale.

In cases where an item does not meet the 80% agreement threshold, it will be considered discrepant. To gain further insights, potential differences in item agreement will be investigated based on factors such as sex, gender, other aspects of diversity, and knowledge of user type. This analysis will be conducted using chi-square analysis.

Content analysis will be carried out for comments received during the survey [68]. These comments will be summarized and used to inform subsequent surveys. The content analysis process will involve the research team and the iKT panel. These results will be summarized and used to inform subsequent surveys. Furthermore, for the rounds 2 and 3 surveys, participants will be given their individual results as well as the overall group distribution, median, and interquartile range from the previous survey rounds (ie, rounds 1 and 2 surveys). These data will be summarized for transparency and to promote full consideration of discrepant items. In addition, as part of this exercise, in the rounds 2 and 3 surveys, we will ask participants to respond to the question, “After reviewing your survey results with respect to this item (ie, each discrepant item), please comment on why you rated this item the way you did.” Again, content analysis will be conducted on these comments to gain additional insights [68].

#### Consensus Meeting

After the completion of the 3 rounds of surveys, a web-based 2-day consensus meeting will be organized using Zoom. All expert participants who participated in rounds 1-3 will be invited to attend this meeting. The meeting will begin with a presentation of the summarized results from round 3, including any accompanying recommendations. We will report back demographic differences. Participants will have the opportunity to elaborate on their ratings of discrepant items.

During the consensus meeting, the researchers and iKT panel will draft the PRECISE guideline. This draft will be presented for discussion. Expert participants will collaborate to reach a consensus (ie, 80% agreement) to determine the items to be included in the final version of PRECISE, as well as determine their specific wording. The meeting will also explore the potential recommendation of a core set of items and the linkage of guideline items to checklist items. If appropriate and feasible, the weighting of items will be considered, and both short and long versions of PRECISE will be discussed.

Additionally, the consensus meeting will address how the PRECISE guideline and checklist can promote diverse perspectives and inclusive reporting. Dissemination strategies for the results will also be discussed during the meeting. Detailed records will be kept of all discussions related to the development of the PRECISE guideline and checklist, documenting the decisions made.

Following the consensus meeting, the guideline and checklist will be distributed to the expert participants for their review and to ensure that it accurately reflects the consensus decisions. The final PRECISE guideline and checklist will be tested with approximately 15 researchers or scientists, policymakers, and students by applying it to a study describing the co-design of a complex health intervention, as done in the development of other guidelines [55].

### Ethical Considerations

Research ethics board approval for this component of the study will be sought by the lead author’s primary institution. PRECISE will seek to reflect not just the prioritized components and associated methods or methodologies of co-design, but also the values or principles that drive co-design and how they can be enacted or enabled.

## Results

This guideline development is supported by the CIHR (Project Grant). This study will take place from April 2023 to March 2025.

## Discussion

### Expected Findings

This research will address the need for consistent reporting in the literature regarding the use of co-design in developing complex health interventions. The guidelines developed through this study will play a crucial role in facilitating future synthesis of studies in this field. The PRECISE guidelines will build on the GRIPP/GRIPP2 guidelines by emphasizing equal partnerships between researchers, practitioners, and knowledge users to generate shared knowledge and solutions. By following these guidelines, researchers can report on the authentic engagement of patients, carers, and other knowledge users, moving beyond thinly described coapproaches to describe methods and techniques that can move the field forward, and consequently lead to enhanced involvement and the promotion of inclusion of vulnerable populations to a greater extent in intervention design. The PRECISE guideline can improve the proliferation of the co-design method by providing researchers seeking grant funding a template to work from, improving reporting, and allowing for refinement of the method itself.

### Strengths and Limitations

This research protocol has several notable strengths that contribute to its methodological soundness and comprehensiveness. One strength of the scoping review is the inclusion of gray literature, which helps mitigate publication bias and ensures a more comprehensive coverage of relevant information [69]. Similarly, we will use an iterative approach to ensure we capture all studies, using guidance from other published reviews and our iKT panel. Additionally, we will conduct a thorough search of reference lists in the included articles and have the search strategy peer-reviewed to further strengthen the quality and comprehensiveness of the search strategy, ensuring that it is thorough and effectively captures relevant literature. All searches will be performed by an information specialist experienced in scoping and systematic review methodologies including reviews on patient engagement. Due to resource limitations, we have made the decision to only include studies that are available in English as part of our inclusion criteria. However, we recognize that this approach may result in the exclusion of relevant studies published in languages other than English. Moreover, our protocol was developed with the EQUATOR guidelines in mind [49].

### Future Directions

We will use a range of passive and active knowledge translation methods to disseminate our research findings. Traditional knowledge translation approaches will involve sharing our findings through local, national, and international meetings, as well as publishing in reputable peer-reviewed journals. Our project will yield open-access publications, including the scoping review itself, and the Delphi process, which will include the PRECISE guideline and checklist along with an explanation and elaboration (E and E) document, consistent with guidance provided by Moher et al [70]. We will widely share the PRECISE guideline and checklist within our networks. Input from our iKT panel and other knowledge users will inform our dissemination strategies and guide the planning of future research initiatives. The research team’s strong connections to key journals in this space, such as *Health Expectations*, *Design for Health*, and the *International Journal of Integrated Care*, highlight our ability to disseminate our contributions to the advancement of co-design methodologies and practices.

### Conclusions

The collective results of this project will lead to the development of a ready-to-implement PRECISE guideline that outlines a minimum set of items to include when reporting the co-design of complex health interventions. The PRECISE guideline will improve the consistency, transparency, and quality of reporting on studies by researchers and multiple knowledge users in policy and practice, including patients, that use co-design to develop complex health interventions. Additionally, it will include guidance on enacting or enabling the values or principles of co-design for meaningful and collaborative solutions (interventions). PRECISE might also be used by peer reviewers and editors to improve the review of manuscripts involving co-design. Ultimately, the PRECISE guideline will facilitate more efficient use of new results in complex health intervention development and bring better returns on research investments.

## Abbreviations

CIHR: Canadian Institutes of Health Research
E and E: explanation and elaboration
EDI: equity, diversity, and inclusion
EQUATOR: Enhancing the Quality and Transparency of Health Research
GRIPP: Guidance for Reporting Involvement of Patients and the Public
iKT: integrated knowledge translation
NIHR: National Institute for Health and Care Research
PPI: patient and public involvement
PRECISE: Preferred Components for Co-Design In Research
PRESS: Peer Review of Electronic Search Strategies
PRISMA-P: Preferred Reporting Items for Systematic Review and Meta-Analysis Protocols
PRISMA-ScR: Preferred Reporting Items for Systematic Reviews and Meta-Analysis Extension for Scoping Reviews
